# A comparison of multiple imputation methods for bivariate hierarchical data: an application to cost-effectiveness analyse

**DOI:** 10.1186/1745-6215-14-S1-O98

**Published:** 2013-11-29

**Authors:** K Diaz-Ordaz, M Gomes, R Grieve, MG Kenward

**Affiliations:** 1 LSHTM, London, UK

## Background

Missing data are common in cluster randomised trials (CRTs) and may lead to biased and inefficient estimation. Multiple imputation (MI) is often used but, to obtain valid inferences, the imputation model must recognise the data structure.

We compare complete case, random–effects MI, fixed-effects MI, and single-level MI, when the analysis model is a linear mixed-model.

## Methods

We begin by illustrating the MI approaches with an example, a cost-effectiveness analysis of a CRT evaluating an intervention for postnatal depression (2659 participants, 100 clusters ICC for cost 0.17, ICC for QALYs 0.04). We conducted a simulation study to assess the performance of the alternative methods. Missing data scenarios were simulated according to factors hypothesized to influence performance, amongst them ICCs, number and size of clusters and the proportion of missing data.

## Results

In the case-study, incremental net benefit estimates (SE) were 81.39 (36.02) for the multilevel MI, 61.72 (36.62) for the fixed effects MI, and 96.00 (50.80) for the single-level MI. In the simulation, complete case resulted in biased estimates (percentage bias between 22% and 60%), while multilevel MI resulted on estimates which were only moderately biased (percentage bias range 0,003%, 3.35%). Fixed effects MI over-estimated the SEs, resulting in CI coverage in excess of nominal levels (up to 100%), whereas multilevel MI reported coverage levels of approximately 95% throughout.

## Conclusion

Estimates may differ depending on how the MI accounted for clustering. Multilevel MI performed well across the settings considered and is appropriate for studies that have a hierarchical design.

